# A report of 2 cases of the use of the Inari FlowTriever System in the treatment of pulmonary embolism

**DOI:** 10.1016/j.radcr.2022.08.081

**Published:** 2022-09-19

**Authors:** Richard Pham, Alexander “Sasha” Ushinsky, Naganathan Mani, Pavan K. Kavali

**Affiliations:** a University of California Riverside School of Medicine, 1550 Central Ave, Riverside, CA 92507, USA; b Mallinckrodt Institute of Radiology, Washington University School of Medicine, St. Louis, MO, USA

**Keywords:** Mechanical thrombectomy, Pulmonary embolism, FlowTriever, Inari

## Abstract

The FlowTriever System (Inari Medical, Irvine, California) is the first FDA-approved mechanical thrombectomy device used for treatment of pulmonary embolism. This device enables nonsurgical removal of pulmonary blood clots without the use of thrombolytic medication and its associated risks. We report 2 cases of successful application of the Inari FlowTriever in treatment of pulmonary embolism and right atrial thrombus.

## Introduction

Conventional treatments of PE include systemic anticoagulation (AC) and catheter-directed thrombolysis (CDT). However, each of these approaches has their own limitations. AC has limited use in cases requiring immediate treatment, as its restorative effects may take as long as 24 hours to be appreciated [1]. CDT, similarly, requires extended tPA infusion times, which may vary from 12 to 33 hours [2]. CDT also has a significant risk of intracranial bleeding and hemorrhage [3]. Furthermore, CDT may be contraindicated in patients whom thrombolytic medication is not advised. Mechanical thrombectomy represents a novel alternative with immediate therapeutic effect and decreased risk of major vessel bleeding.

## Case presentation

### Case 1

A 62-year-old female presented to the emergency department with chest and shoulder pain after a syncopal fall. She has a past medical history of diabetes mellitus type 2, hypertension, and rheumatoid arthritis. Presentation occurred prior to the onset of the COVID-19 pandemic.

**Fig. 1:**
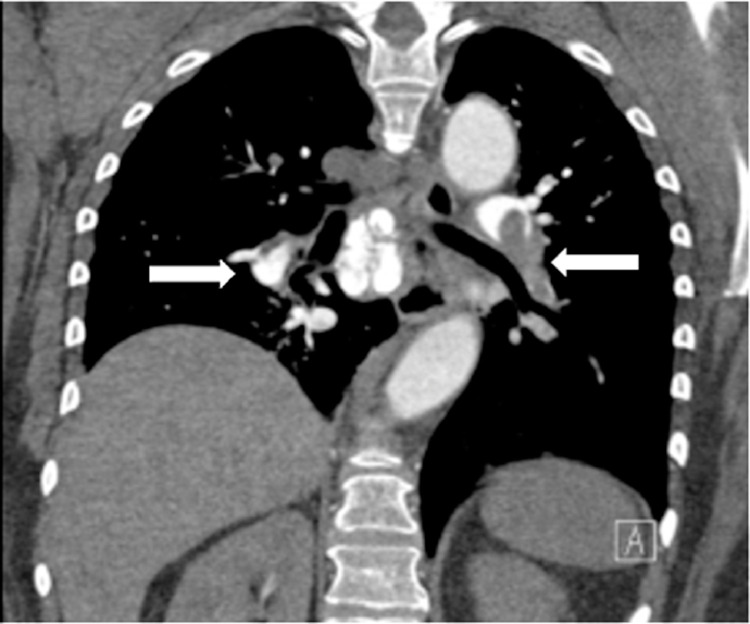
(Case 1). CT imaging demonstrated bilateral segmental and subsegmental pulmonary emboli within the bilateral upper, middle, and lower lobe (arrows).

Chest CT demonstrated bilateral segmental and subsegmental pulmonary emboli within the bilateral upper, lower, and middle lobe (Fig. 1). There is a central saddle embolus with evidence of right heart strain. A clot within the right atrium extending into the right ventricular outflow tract was also appreciated, likely representing a clot in transit. Flolan and Heparin drip were immediately started. Echocardiogram revealed left ventricular ejection fraction of 66%, mild tricuspid regurgitation, and a large, mobile thrombus in the right atrium extending into the right ventricular outflow tract. The Inari FlowTriever (IFT) 24-French system was advanced and the Triever24 large lumen catheter was advanced with the tip abutting the thrombus in the right lower, right upper, and right pulmonary arteries. A moderate clot burden was successfully aspirated. Attempted aspiration of the clot was performed in the right atrium. However, intracardiac echocardiography and repeat right pulmonary angiogram demonstrated that the attempted thrombectomy had caused the right heart thrombus to eject into the right ventricular outflow tract, causing decreased perfusion to the right lung. The technique was repeated unsuccessfully in the right main pulmonary artery. Because the clot was too large to be aspirated, 6 mg of tPA was injected in the right main pulmonary artery to aid in the lysis of the clot.

**Fig. 2:**
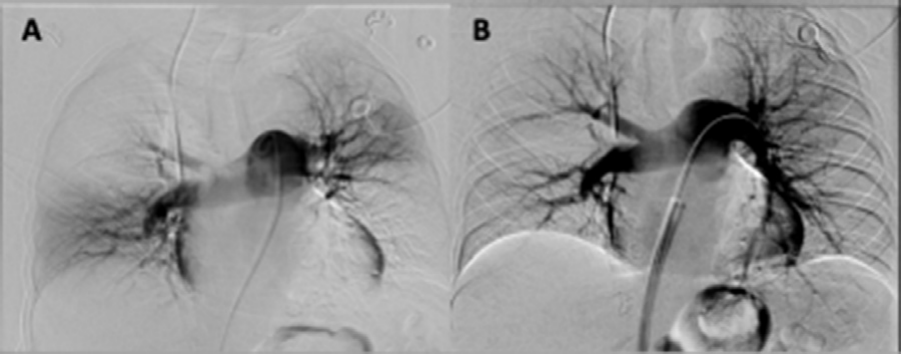
(Case 1). (A) Pre-PA Angio; (B) post-PA Angio.

The patient decompensated post procedurally, and was put on extracorporeal membrane oxygenation and maximum vasopressor support. Follow up chest CT demonstrated saddle pulmonary embolus with thrombus extending into multiple segmental subsegmental pulmonary arteries. Using the same technique, a large clot burden was successfully aspirated from the left lower lobe pulmonary artery and the right main pulmonary artery. Final pulmonary angiogram revealed successful removal of the clot and increased perfusion (Fig. 2). Pre thrombectomy pressure of the main pulmonary artery was 48/18 (mean of 29) mmHG, while post-thrombectomy pressure was 40/10 (mean of 19) mmHG. The patient subsequently stabilized and was offered post-procedure supportive care. The patient was discharged without further incident.

### Case 2

A 78-year-old female presented with postoperative complications of hypotension and bradycardia 5 days after a cervical laminectomy procedure. She has a past medical history of diabetes mellitus type 2, hypertension, and cervical myelopathy. Presentation occurred prior to the onset of the COVID-19 pandemic.

**Fig. 3:**
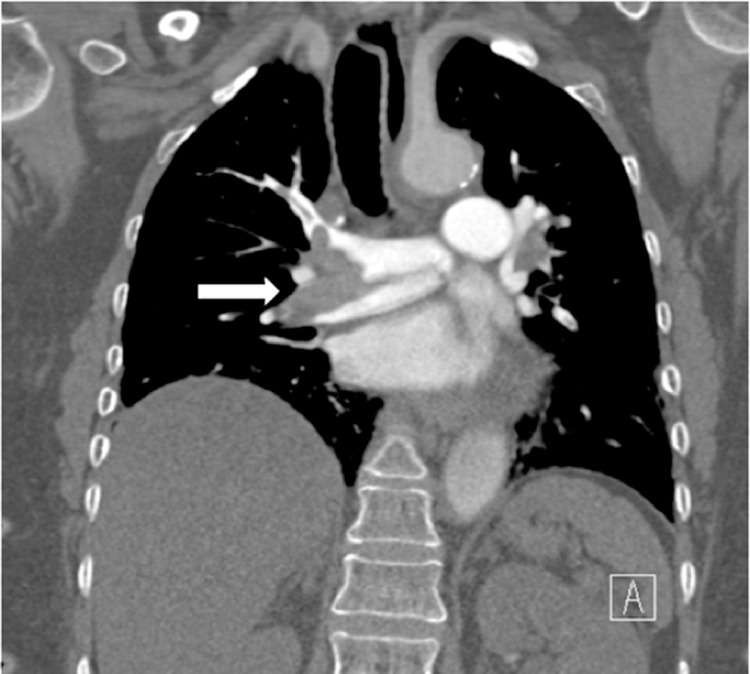
(Case 2). Chest CT demonstrated acute pulmonary embolism with saddle embolus in the right pulmonary artery (arrow).

Bedside echocardiogram demonstrated a suspected clot in transit in the right atrium. Chest CT demonstrated acute pulmonary embolism with saddle embolus in the right pulmonary (Fig. 3). Multiple additional pulmonary emboli were also observed throughout the bilateral upper and lower lobes with evidence of right heart strain. Due to her increased risk of postoperative bleeding, the patient was not a candidate for therapeutic systemic lytic therapy. A Pulmonary Embolism Response Team (PERT) consensus decision was reached to proceed with mechanical thrombectomy.

**Fig. 4:**
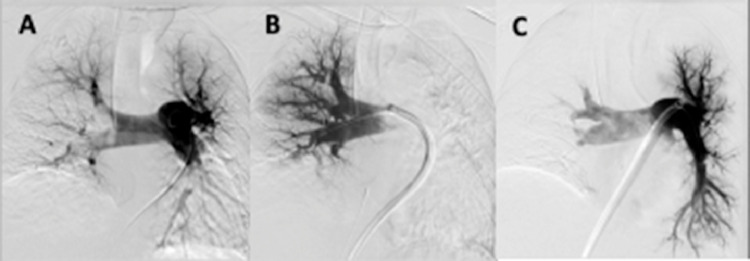
(Case 2). (A) Pre-PA Angio; (B) RPA post Angio; (C) LPA post Angio.

The IFT 20-French system and the Treiver20 large lumen catheter were advanced. With the tip abutting the thrombus in the right atrium, a large clot burden was successfully aspirated. The technique was repeated successfully in the right lower pulmonary artery, the right upper lobe pulmonary artery, and the left main pulmonary artery. Finally, a Denali filter was deployed in the infrarenal IVC. Pulmonary angiogram demonstrated no residual clot (Fig. 4). Prethrombectomy pressure of the main pulmonary artery was 41/21 (mean of 29) mmHG, while post-thrombectomy pressure was 32/20 (mean of 23) mmHG. The patient subsequently stabilized from both respiratory and cardiovascular perspectives. The patient was offered post-procedure supportive care and was later discharged without further incident.

### Ethics approval

The information provided for the above submission was evaluated and determined to not meet the definition of human subjects research. Accordingly, IRB approval and continuing review is not required.

## Discussion

Anticoagulation medication has long been the mainstay of treatment for the majority of pulmonary emboli. However, complex cases of larger emboli often require a more aggressive, immediate approach. Furthermore, thrombolytic medication can be contraindicated in a variety of circumstances, including patients who have been diagnosed with recent head trauma, recent stroke, brain neoplasms, or pregnancy [4]. Nearly 30% of the PE population are postoperative patients who are at high risk of bleeding [5]. Mechanical thrombectomy represents an alternative approach that can be utilized in conjunction with anticoagulation therapy or as an independent treatment modality. RV/LV diameter ratio is the strongest predictor of short-term mortality and negative outcomes [6]. As such, the RV/LV ratio has become the major biomarker for evaluating case severity and treatment success. The FLARE (FlowTriever Pulmonary Embolectomy Clinical) study reports that use of IFT resulted in a RV/LV ratio reduction of .38, while CDT resulted in a RV/LV ratio reduction of 0.34 [3]. Another single-institution review reported IFT use resulted in 100% technical and 88% clinical success, with a 71% improvement in supplemental oxygen requirements [7].

Furthermore, use of IFT has a much lower major complication rate in comparison to CDT. CDT is associated with major bleeding in 9.3% of patients, with intracranial hemorrhage affecting 1.5% of those instances [8]. In comparison, the FLARE study reported a 0.9% major bleeding rate with no instances of intracranial hemorrhage [3]. The FLARE study also reported a similar clinical deterioration rate to both CDT and anticoagulation therapy. As such, use of the IFT system is appreciably safer than both CDT and anticoagulation therapy.

## Conclusion

Mechanical thrombectomy represents a safe and effective alternative to anticoagulation therapy and catheter directed thrombolytics.

## Patient consent

Written and informed consent was obtained for publication from the patient.

## Compliance with ethical standards

For this type of study, formal consent is not required.

For this type of study, informed consent is not required.
